# Effect of Different Flooring Designs on the Performance and Foot Pad Health in Broilers and Turkeys

**DOI:** 10.3390/ani8050070

**Published:** 2018-05-03

**Authors:** Bussarakam Chuppava, Christian Visscher, Josef Kamphues

**Affiliations:** Institute for Animal Nutrition, University of Veterinary Medicine Hannover, Foundation, Bischofsholer Damm 15, D-30173 Hanover, Germany; bussarakam.chuppava@tiho-hannover.de (B.C.); josef.kamphues@tiho-hannover.de (J.K.)

**Keywords:** broiler, turkey, flooring design, slatted flooring, floor heating, performance, foot pad dermatitis

## Abstract

**Simple Summary:**

The contact of the birds’ feet with litter and their excreta during the fattening period might lead to reduced body weight, carcass weight, feed intake, and impaired foot pad health in poultry. This study was performed to evaluate the influence of different flooring designs with reduced (50% or 100% slatted floors) contact to the excreta on the above-mentioned parameters in the fattening of broilers and turkeys. The provision of litter on the floor had no relevant effect on foot pad health in broilers. Using fully-slatted flooring in fattening turkeys led to a higher body weight, while reducing incidence of injuries of foot pads. It is, therefore, necessary to consider how a similar excellent litter quality can be achieved in basically littered husbandry systems common in Europe.

**Abstract:**

Litter quality has a significant influence on the performance and foot pad health in poultry. The objective of this study was to evaluate the effects of different types of flooring designs on the performance and foot pad health in fattening broilers and turkeys. Three trials were conducted for each species using a total of 720 Ross 308 broilers and 720 Big 6 turkeys. After day seven, animals were randomly assigned to four groups with three subgroups each: G1—floor pens with litter, G2—floor pens with litter and floor heating, G3—partially-slatted flooring, including a littered area, and G4—fully-slatted flooring with a sand bath (900 cm^2^). Animals of both species had a significantly higher final body weight at dissection (day 36) after being reared on fully-slatted floors compared to common littered floors. In turkeys, the feed conversion ratio was worse in G4 (1.53 ± 0.04) than in G1 (1.47 ± 0.02) and G2 (1.48 ± 0.03). Water to feed ratio was significantly higher in G2 than other groups. Turkeys’ foot pad health was significantly better in G4 than in other groups beginning at day 21. In turkeys, platforms with slatted floors that allow for temporary separation of the feet from the litter could lead to improvements in foot pad health which could better enable the realization of species-specific behaviours and activities in littered areas.

## 1. Introduction

Housing and management are significant factors regarding animal health and welfare in poultry. Housing of poultry on littered concrete floors is the common system for commercial poultry production in Europe. In particular, the litter is also there to satisfy the birds’ need for pecking, scratching, and dust bathing, which should be considered essential for the welfare of the animals [1,2]. At the end of the fattening period, approximately 80% of the dry matter (DM) of the total litter material are due to excreta and feed residues [3]. Consequently, poultry is continuously in contact with litter consisting mainly of their excreta. If drying properties are poor, litter conditions become suboptimal. Therefore, it can be expected that birds will develop contact dermatitis with ulcerations of the skin affecting the plantar surface of the feet (foot pad dermatitis; FPD), the hock (hock burn), and the breast (breast irritations) [4,5,6]. In addition, the incidence and severity of contact dermatitis may be used as a welfare assessment measure in commercial poultry production systems. There is evidence that the severe FPD causes pain and, thus, is a matter of animal welfare [1,7,8].

For more than 30 years, researchers have been investigating the impact of litter quality on the foot pad health and performance of different poultry species, including broilers [9,10,11] and turkeys [3,12,13,14]. Several studies suggested that there is a strong association between poor litter conditions and foot pad dermatitis [15,16,17,18]. The prevalence of FPD in broilers and turkeys is closely related to high concentrations of litter moisture [19]. High litter moisture alone without the presence of excreta was sufficient to cause FPD in both species [17,20]. FPD is a widespread challenge in broiler and turkey production and is a potential economic and welfare issue in intensive production systems. The prevalence and severity of FPD is an issue of importance to the poultry industry, with up to 98% of turkeys being affected [21,22]. Serious lesions of the foot pads can be very painful and cause stress [23], and might result in the appearance of other infections causing deterioration in general health conditions. Reduced performance, including poorer FCR, and body weight gains and lower final body weights are the result [15].

Litter quality is affected by many variables, such as humidity, season, drinker design, amount and consistency of excreta [24], and high stocking density [25], the excreta quality often being related to dietary factors [3,26]. Various nutrients can additionally affect the foot pad health, such as unbalanced levels of protein, minerals, or vitamins [27,28,29,30]. Other factors are linked to management and housing [4,13,17]. Therefore, focusing on dietary strategies, as well as management conditions that affect performance in the field are of importance. The role of management in reducing litter moisture and decreasing FPD, as well as using floor heating for improving litter quality resulted in significantly lower FPD scores compared to foregoing floor heating [13,31]. Moreover, studies with rearing broilers and turkeys on flooring other than litter have increased for economical and managerial reasons in the commercial poultry industry. Poultry farming with slatted flooring in parts of the flock is already used in the breeding of laying hens [32] and has recently been used in meat-producing chickens in some parts of South Asia and Russia [33]. This is always done to reduce the contact between birds’ feet and their excreta. The designs of flooring without litter would imply a behavioural restriction for the birds and could have a negative effect of animal welfare. However, so far, studies have mainly focused on the effect of flooring design with regard to production performance and foot pad health in the fattening of broilers and turkeys [34,35,36].

The objective of this study was to evaluate the effects of using different flooring designs on performance and foot pad health. Therefore, littered flooring with or without floor heating was compared to systems with reduced (50% or 100% slatted floors) contact to the excreta.

## 2. Materials and Methods

The experiments were performed in accordance with German regulations and approved by the Ethics Committee of Lower Saxony for Care and Use of Laboratory Animals LAVES (Niedersächsisches Landesamt für Verbraucherschutz und Lebensmittelsicherheit; reference: 33.12-42502-04-15/2044).

### 2.1. Housing and Experimental Design

Six consecutive trials with 240 birds each were conducted: three experiments starting with one-day-old broiler chickens (as hatched; Ross 308; N = 720) and three experiments with female turkeys (BUT-Big 6; N = 720). Broilers and turkeys had been obtained from commercial hatcheries (BWE-Brüterei Weser-Ems GmbH and Co. KG, Visbeck, Germany and Heidemark GmbH, Ahlhorn, Germany, respectively). The birds were housed in floor pens prepared with wood shavings (GOLDSPAN^®^, Goldspan GmbH and Co. KG, Goldenstedt, Germany) for the first seven days. Litter material was kept dry and clean before the experiment was started by removing the upper layers of the litter daily and replacing them with fresh dry litter. All birds were fed ad libitum with commercially-prepared pelleted diets (Best 3 Geflügelernährung GmbH, Twistringen, Germany, Table 1). After a one-week adaptation period, each experiment was started. The birds were divided into four groups, each having three identical subgroups (*n* = 20 birds). At the end of day 21 in each subgroup of 20 animals, eight birds were dissected. As anesthesia or rather stunning method in accordance with Annex I of Council Regulation (EC) No. 1099/2009, Chapter I, Methods, Table 1—Mechanical Methods [37], No 6, a percussive blow to the head was made. After bleeding the animals the body cavity was opened, the sternum lifted up and the gastrointestinal tract and the other organs of the corpuscle (liver, gall-bladder, spleen, and heart) were also removed and the head and legs in the tarsal joint were truncated. The feathers were not removed. All remaining turkeys (*n* = 12/box) were dissected at day 36 by the same method.

Twelve experimental pens (1.20 × 0.80 m) were placed in a randomised sequence of four subgroups (G1–G4) in three blocks in the same stable. A vacuum air ventilation system was installed in the ceiling in two rows above the pens. The pens were bedded with approximately 1 cm (1 kg per square metre) of wood shavings without removing litter during the experiments as it was done in the adaptation phase. Stocking densities reached about 35 kg per square metre for broilers and 25 kg per square metre for turkeys at the end of the trial. The slatted floor consisted of holes (15 × 10 mm) and bridges (plastic covered steel; width 3.5 mm; Big Dutchman International GmbH, Vechta, Germany). The excreta were stored during the entire fattening period under the slatted flooring at a depth of approximately 30 cm. Housing systems were identical for both species. On the left-hand side of each pen, there was a scratching area (SA), and on the right-hand side there was a feeding area (FA) equipped with one hanging-type feeder (Crown Chicken Ltd., Norwich, UK). Two different drinking water systems were used with nipple drinkers for broilers (Big Dutchman International GmbH, Vechta, Germany) and bell drinkers for turkeys (Ferdinand Stükerjürgen GmbH & Co. KG, Rietberg-Varensell, Germany). The birds had ad libitum access to fresh, clean water and a commercial pelleted growing diet. The environmental temperature was gradually reduced from about 33 °C for the one-day-old birds to about 20 °C by day 36. Lights were continuously on at days 1, 2, and 3 and the photoperiod from day 4, onwards, was 16 h of light and 8 h of darkness.

**Figure 1:**
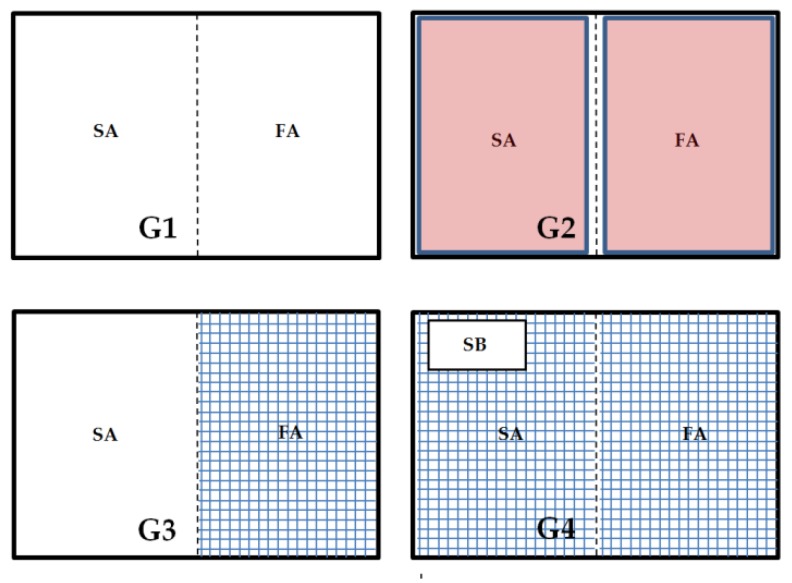
Characteristics of flooring designs: G1 = entire floor pen covered with litter (wood shavings); G2 = floor pen covered with litter and having floor heating (in red); G3 = partially (50:50)-slatted flooring (in blue) including an area with litter; G4 = fully-slatted flooring with a sand bath (900 cm^2^). SA = scratching area, FA = feeding area, SB = sand bath.

In the groups, different flooring designs were used to establish different degrees of contact intensity of the animals to the excreta (Figure 1). The first group was kept on dry wood shavings (G1—entire floor pen covered with litter); the second group was kept on dry wood shavings, but also with floor heating (G2—floor pen with litter with floor heating). An electrical floor heating system supplied with an adjuster to control the temperature was used (Sauerland GmbH, Paderborn-Elsen, Germany). The birds in G1 and G2 had full contact with the mix of excreta and wood shavings during the entire study period. The third group (G3) was housed in a floor pen that was divided into two equal parts consisting of 50% wood shavings on the scratching area (left-hand side) and 50% plastic slatted flooring on the feeding area (right-hand side). The fourth group (G4) was housed completely on plastic slatted flooring with a sand bath (900 cm^2^). Animals in G4 had the lowest contact intensity with litter except possibly within the sand bath.

### 2.2. Measurements

#### 2.2.1. Technical Performance

The individual body weight (BW) was measured weekly on the same day. At dissection (day 36) final body weight, as well as carcass weights (eviscerated, without head and feet) were monitored. Feed (FI) and water intakes (WI), as well as losses were determined at subgroup level. Feed conversion ratio (FCR) and water to feed ratio (W:F-ratio), were estimated on the basis of feed and water consumed (data from groups) throughout the experimental period.

#### 2.2.2. Foot Pad Dermatitis Scoring Criteria

**Figure 2:**
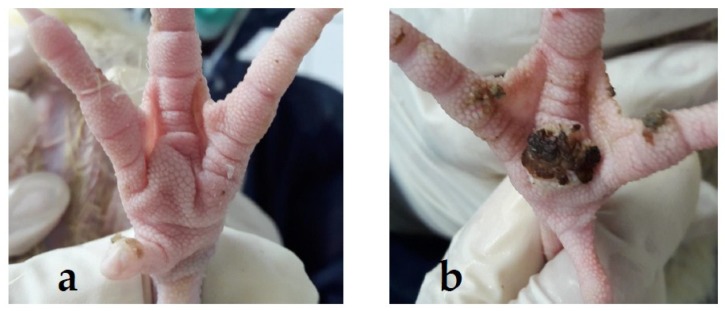
Photograph of foot pad lesions with different scores. (**a**) Category 0: no alterations detected; and (**b**) Category 4: more than half of the foot pad covered with necrotic cells (photo: © Chuppava/TiHo).

The external examination of foot pads was performed for all birds at the beginning of the experiment (day 7), then weekly until day 35. If the feet were dirty, they were carefully washed with wet cloth to remove slightly adhering litter and excreta from the feet. Foot pads were dried with tissue paper before scoring. The central plantar was scored. Signs of foot pad lesions were recorded on a five-point scale; five categories ranging from 0 (unaffected—no external signs of FPD) to 4 (more than half the foot pad covered with necrotic cells) in accordance with Hocking et al. [38] (Figure 2). Other measures concerning behaviour, use of space, use of the sandbox, and other welfare indicators might have been very useful, but were not part of the study. They should be considered in further studies.

#### 2.2.3. Litter Measurements

Litter samples for measuring the dry matter (DM) content were collected weekly from two defined locations: the feeding and the scratching area in each pen. At each area, a sample (50 g) from three sites (two peripheral samples and one central one) over the whole bedding height was punched out using a cup with a diameter of 5 cm from the full depth of the litter. Samples were dried at 103 °C for the time needed to reach a constant weight.

### 2.3. Statistical Analysis

The data analyses were performed using the SAS statistical software package version 7.1 (SAS Inst., Cary, NC, USA). Mean values, as well as the standard deviation of the mean (SD), were calculated for all parameters, as well as individual BW and carcass weights. The mean FPD-scores were evaluated by using the mean of both feet. The feed and water intakes, FCR, W:F-ratio, and percent DM content in litter were estimated at the pen level, as well as the final BW and FPD for correlation analysis. For the description of the prevalence of FPD, two-dimensional frequency distributions of categorical features were checked for dependency by means of the Pearson‘s chi square homogeneity test.

The group comparisons were performed by one-way analysis of variance (ANOVA) for independent samples. The Ryan-Einot-Gabriel-Welsch multiple range test (REGWQ) was used for multiple pairwise means comparisons between the four groups of flooring design. A Pearson’s correlation coefficient was calculated to evaluate the relationship between the final BW and final FPD-scores at the pen level between groups. All statements of statistical significance were based on *p* < 0.05.

## 3. Results

### 3.1. Growth Performance

The results related to growth performance of broilers and turkeys are shown in Table 2. In broilers, after 35 days of fattening, BW exceeded the performance goals of the breeding company (Table 2; [39,40]). BW showed no significant differences between groups in both species before starting the trials. Additionally, during the trial there were no significant differences in BW (BW at day 35; *p* < 0.08) in broilers between the groups. At dissection the average final BW for broilers in groups with a partly- or fully-slatted floor was significantly higher (in g; G1:2555^b^, G2:2570^b^, G3:2655^a^, G4:2698^a^; Table 2).

For turkeys kept on fully-slatted flooring, there was a significantly higher average BW from day 21 onwards up to dissection (Table 2). As a consequence, turkeys reared on fully-slatted flooring had a higher final live weight (G4: 2087 ± 171 g) than birds in G1 (1990 ± 185 g). The weight of the eviscerated carcass in this group was also significantly higher (in g; G1:1545^b^, G2:1532^b^, G3:1567^b^, G4:1612^a^) than that of the other groups. Furthermore, feed intake was simultaneously higher in G4 for turkeys than in other groups (Table 3).

No differences were observed concerning FI and FCR in broilers kept on different flooring designs (Table 3). Turkeys kept on partial- and fully-slatted flooring were characterised by higher FI and FCR than the birds housed on a litter system. In both species, the water to feed ratio was significantly higher when using litter floor pens with floor heating. The average floor surface temperature was highest in group G2 in all pens (in °C; G1 = 27.0, G2 = 30.5, G3 = 26.5, G4 = 26.0).

### 3.2. Foot Pad Dermatitis

In broiler trials there were no clinical issues concerning foot pad health. Nonetheless, broiler chickens kept on wood shavings without or with floor heating (G1 and G2) were characterised by significantly lower FPD-scores in the five-point scale in comparison to birds in G4 on day 14 (Table 4). On analysing the frequency of foot pad lesions (Table 5), it was found that, at the end of the broiler trials, 100% of the animals had FPD-scores ≤ 1 and no significant differences between groups.

Turkeys generally had poorer foot pad health than broilers. This means scores were higher. Turkeys kept on fully-slatted floors had significantly lower foot pad scores than those reared on littered or partly-slatted floors. FPD-scores were significantly lower when using a slatted floor in the period from day 28 up until the end of the trial compared to the other flooring designs (Table 4). In the case of turkeys, regardless of the flooring system, nearly 100% of the observed turkeys showed a clinically-apparent FPD at the end of the trial. The majority of birds showed intermediate foot pad lesions (score 2). The prevalence of severe FPD lesions was significant lowest in group G4 (4%).

**Figure 3:**
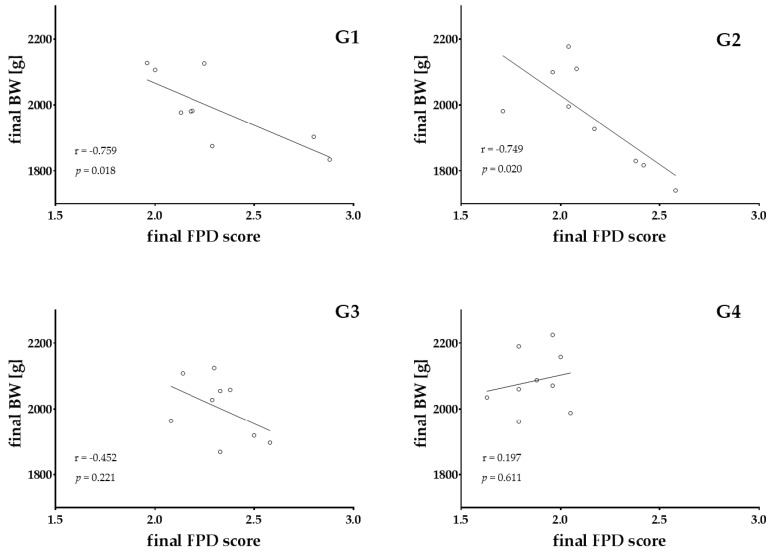
Spearman’s rank correlation coefficients with significance levels between the final body weight and final FPD-scores in the turkey experiments are displayed (*p* < 0.05). G1 = entire floor pen covered with litter; G2 = floor pen covered with litter and having floor heating; G3 = partially (50:50)-slatted flooring including an area with litter; G4 = fully-slatted flooring with a sand bath (900 cm^2^).

Figure 3 shows the Spearman’s rank correlation coefficient between the final BW and final FPD-scores in turkey experiments. In G1 and G2, the correlations between both parameters were significantly highly negative (r = −0.759 and r = −0.749, *p* < 0.05, respectively). With slatted systems, a correlation between body mass and foot pad health did not exist.

### 3.3. Litter Quality

**Figure 4:**
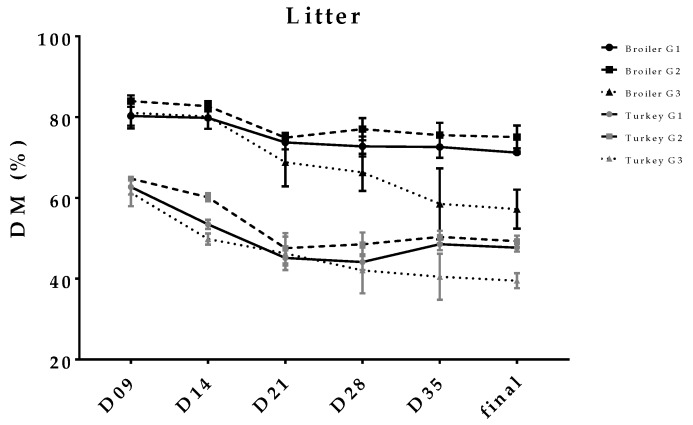
DM content (%) of the litter mixture of excreta in pens with different flooring designs during the experimental period.

In Figure 4, only the values from litter in the scratching area are listed. Group G2 was characterised by the highest content of dry matter (lowest moisture content), whereas the lowest values were seen in groups with partially-slatted flooring. The litter quality of the final litter was significantly worse in turkeys than in broilers; the average final DM in broiler trials: G1 = 71.2, G2 = 75.1, G3-litter area = 57.2% and in turkey trials: G1 = 47.7, G2 = 49.3, G3-litter area = 39.5%.

## 4. Discussion

For poultry to be able to perform to their expected growth performances they should be reared with good management and environmental conditions, including optimal litter quality and housing systems. However, performance is only one criterion. At least as important, birds also need to be able to show their normal behaviour, including ground pecking, scratching, and dust bathing. The outcome of the current study showed that broilers and turkeys given identical dietary regimens in slatted flooring system had a significantly higher final BW compared with those housed in litter systems. Specifically, the final BW was 143 g and 97 g higher for broilers and turkeys, respectively, reared on fully-slatted flooring than those reared in litter floor pens. Similar results were obtained from Almeida et al. [35] and Çavuşoğlu et al. [36]. Broilers reared on plastic slatted flooring had relatively higher weight gains (+8.10% for the final BW in the latter named study) and a higher FCR than observed for chickens reared on wood shavings. Slatted flooring might offer almost no possibilities for the birds to peck and manipulate particles when no litter particles are available on the ground, therefore, feed pecking occurs rather than pecking at the slatted floor [41] resulting in higher feed intake. Pereira et al. [42] observed in the slatted flooring system that there was air movement in the plenum between the manure and the perforated floor, and increased air movement can reduce heat stress in broilers. Heat stress negatively affects the welfare and productivity of broilers [43]. In this study, in broilers there were no clinical problems with foot pad health in all floorings systems. This is different from a previous study in broilers raised in litter floor pens [44]. In the named study living directly in contact with faeces for a long period led to a higher incidence of foot pad inflammation. This might reduce the weight gain during later phases. In contrast, Li et al. [34] showed that performance was not affected by the plastic perforated flooring compared to the litter system in broiler production.

On the basis of data given in the literature, litter moisture is important for the prevalence and severity of foot pad alterations [3]. Mayne et al. [17] suggested that wet litter alone may be the cause of FPD in turkeys. Therefore, reducing the litter moisture by using floor heating or minimizing contact with wet litter and excreta by using slatted flooring is expected to lead to significant improvements in FPD. In this study, the results for both species from experiments conducted with different floor temperatures were insufficiently conclusive to be able to show whether litter floor pens with floor heating were superior to an entire floor pen without floor heating. Contrary to the present results, Abd El-Wahab et al. [13] stated that birds housed on floor heating showed significantly lower external FPD scores compared to poultry in groups without floor heating. The effect of using floor heating on FPD scores could be due to fresh litter becoming dry or to floor heating leading to warm foot pads causing vasodilation of the blood vessels, increasing the blood flow [7]. In the current study, however, the temperature of the bedding was higher compared to other pens without floor heating, but the litter itself was not significantly drier. This may be related to another observation as broilers and turkeys reared on floor heating had a significantly higher W:F ratio in this study. Water intake (WI) generally increases at high environmental temperatures [45]. Furthermore, it was observed that the increases in WI was also reflected in a progressive increase in litter moisture [30]. Despite forced WI in the floor heating group in this study, the litter moisture contents in the floor heating group and that without floor heating resulted in scarcely different values. The floor heating used in the present study was not sufficiently efficient to generate drier litter.

After 35 days of fattening, 100% of the broilers had a FPD score ≤ 1 despite the high stocking density of about 35 kg/m^2^, indicating that the floor material without litter did not harm broilers’ foot pads. The results of this study show neither negative nor positive effects for one of the different floor designs on FPD and performance in broilers. Çavuşoğlu et al. [36] showed a lower frequency of FPD in birds raised on slatted flooring. On the other hand, Almeida et al. [35] demonstrated a slight tendency towards higher FPD scores for birds housed on slatted flooring.

To the best of our knowledge, the effect of flooring design-related differences on foot pad health in the fattening of turkeys has not been previously reported. The results from this study suggested that, as expected, minimising contact between turkeys’ feet and their excreta by using fully-slatted flooring showed better foot pad health than the other flooring designs. Martland et al. [7] and Ekstrand and Algers [21] proved that poor litter management increased the prevalence and severity of FPD in turkeys, as well as leading to lower weight gains. Similar to the results of this study for littered systems, performance was negatively correlated with the FPD scores in turkeys. It is possible that turkeys with high FPD scores had a decreased body weight. This has been suggested to be a result of pain-induced lowered feed intake [23] which, in turn, leads to a reluctance to move and, thus, decreased feed consumption and impaired product quality [46]. On the other hand, in the present study, when turkeys were housed on slatted flooring, there was no correlation between FPD and body weight. Body weight of birds with higher scores was only numerically higher. Da Costa et al. [47] indicated that higher FPD scores might be more related to mechanical pressure. Increasing mechanical pressure from higher body weight could lead to decreased mobility and lower feed intake, also affecting animal welfare. However, more research should be conducted to study the effects of slatted flooring on poultry welfare: behaviour, use of space, use of the sand bath, and other welfare indicators might be very useful.

## 5. Conclusions

Litter quality has a significant influence on performance and foot pad health in fattening broilers and turkeys. The results in this study do not justify the use of slatted flooring systems. However, minimising the contact between birds and poultry manure seems to favour the performance and foot pad health in turkeys. Overall, the broiler husbandry conditions were good in that no relevant differences could be identified. Water and feed consumption per kg of meat in turkey production was higher than in broiler production. It can be suggested that fully-slatted areas could be an interesting added enrichment tool in turkey production. However, the results clearly show that identical stocking density combined with partially-slatted flooring at a ratio of 50:50 to the littered area offers no advantages for turkeys. Due to a certain preferential behaviour of the animals to the littered area, the litter there is disproportionately poor, which compensates for the possible advantages of the perforated areas. Therefore, slatted areas should be made more attractive to turkeys by adding elevated platforms as an additional offer or placing feeding and drinking resources in this area. Increasing the amount of time turkeys spend in these new areas could result in separating parts of the excreta from the animals leading to drier litter areas. This could give the littered areas an added value for the realization of natural behaviours in birds. Together, this could benefit their health and performance, too.

## Figures and Tables

**Table 1:** Analysed nutrient contents of the diets during the whole experimental period (g/kg as fed).

Chemical Composition (g/kg)	Broiler Diet			Turkey Diet	
	Starter	Grower	Finisher	Starter P0	Starter P2
Dry matter (DM)	883	878	874	888	890
AME_N_ * (MJ/kg)	12.2	12.4	12.6	10.9	11.7
Crude protein (CP)	217	203	205	278	285
Na	1.30	1.26	1.32	1.61	1.44
K	7.51	7.08	6.73	12.3	12.2

* AME_N_ (pe kg) = 0.1551 × %CP + 0.3431 × %EE + 0.1669 × %starch + 0.1301 × %sugar (as sucrose).AME_N_ = nitrogen-corrected apparent metabolizable energy; EE = Ether extract.

**Table 2:** Development of body weight in fattening broilers and turkeys reared on different floor designs (mean ± SD).

Species	Group	Day/Body Weight [g]						Carcass [g]
		7	14	21	28	35	Final (36)	
Broiler [*n* = 720; *n* = 429 *]	1	198 ± 22.5	528 ± 61.4	1066 ± 127	1757 ± 188	2479 ± 269	2555 ^b^ ± 283	2091 ± 229
	2 **	198 ± 21.0	528 ± 56.6	1060 ± 130	1760 ± 1167	2486 ± 247	2570 ^b^ ± 258	2097 ± 210
	3	198 ± 20.7	535 ± 51.4	1075 ± 108	1779 ± 163	2532 ± 243	2655 ^a^ ± 255	2114 ± 221
	4 **	198 ± 19.3	540 ± 51.5	1086 ± 116	1790 ± 148	2554 ± 247	2698 ^a^ ± 266	2162 ± 206
Turkey [*n* = 720; *n* = 431 *]	1	173 ± 13.7	388 ^bc^ ± 37.5	705 ^b^ ± 72.0	1213 ^bc^ ± 94.0	1907 ^b^ ± 174	1990 ^b^ ± 185	1545 ^b^ ± 165
	2	172 ± 14.9	383 ^c^ ± 37.2	708 ^b^ ± 71.9	1198 ^c^ ± 111	1884 ^b^ ± 197	1964 ^b^ ± 215	1532 ^b^ ± 189
	3	175 ± 14.2	395 ^ab^ ± 35.6	720 ^b^ ± 73.2	1233 ^b^ ± 104	1924 ^b^ ± 174	2003 ^b^ ± 169	1567 ^b^ ± 158
	4 ***	174 ± 14.3	400 ^a^ ± 39.4	745 ^a^ ± 85.3	1307 ^a^ ± 116	1996 ^a^ ± 169	2087 ^a^ ± 171	1612 ^a^ ± 176

^a, b^ means in the same column at species level, values with different superscript letters mean significant differences between groups (*p* < 0.05). * number of animals after first dissection, ** two animals died, *** one animal died. G1 = entire floor pen covered with litter; G2 = floor pen covered with litter and having floor heating; G3 = partially (50:50)-slatted flooring including an area with litter; G4 = fully-slatted flooring with a sand bath (900 cm^2^).

**Table 3:** Feed intake (FI), feed conversion ratio (FCR), and water to feed ratio (W:F ratio) during the experimental period (day 8–day 36) in broilers and turkeys reared on different floor designs (mean ± SD).

Species	Group	FI [g]	FCR	W:F Ratio
Broiler [*n* = 720; *n* = 429 *]	1	3604 ± 97.8	1.53 ± 0.06	1.85 ^b^ ± 0.03
	2 **	3624 ± 118	1.53 ± 0.08	2.02 ^a^ ± 0.02
	3	3663 ± 61.5	1.49 ± 0.03	1.86 ^b^ ± 0.02
	4 ***	3698 ± 145	1.48 ± 0.09	1.84 ^b^ ± 0.04
Turkey [*n* = 720; *n* = 431 *]	1	2668 ^b^ ± 170	1.47 ^b^ ± 0.02	2.62 ^b^ ± 0.10
	2	2656 ^b^ ± 205	1.48 ^b^ ± 0.03	2.79 ^a^ ± 0.11
	3	2737 ^ab^ ± 132	1.50 ^ab^ ± 0.03	2.53 ^b^ ± 0.10
	4 ***	2883 ^a^ ± 167	1.53 ^a^ ± 0.04	2.65 ^b^ ± 0.14

^a, b^ means in the same column at species level, values with different superscript letters mean significant differences between groups (*p* < 0.05). * number of animals after first dissection, ** two animals died, *** one animal died. G1 = entire floor pen covered with litter; G2 = floor pen covered with litter and having floor heating; G3 = partially (50:50)-slatted flooring including an area with litter; G4 = fully-slatted flooring with a sand bath (900 cm^2^).

**Table 4:** Development of external foot pad scores (FPD) in broilers and turkeys kept on four different flooring designs throughout the experimental period (mean ± SD).

Species	Group (*n* = 9)	Day/FPD-Scores				
		7	14	21	28	35
Broiler	1	0.06 ^a^ ± 0.08	0.02 ^a^ ± 0.04	0.09 ^a^ ± 0.07	0.22 ^a^ ± 0.17	0.40 ^a^ ± 0.24
	2	0.04 ^a^ ± 0.04	0.02 ^a^ ± 0.02	0.09 ^a^ ± 0.08	0.30 ^a^ ± 0.18	0.45 ^a^ ± 0.26
	3	0.04 ^a^ ± 0.04	0.07 ^ab^ ± 0.07	0.08 ^a^ ± 0.08	0.41 ^a^ ± 0.26	0.64 ^a^ ± 0.20
	4	0.03 ^a^ ± 0.02	0.10 ^b^ ± 0.10	0.13 ^a^ ± 0.11	0.42 ^a^ ± 0.26	0.59 ^a^ ± 0.27
Turkey	1	1.00 ^a^ ± 0.07	1.05 ^a^ ± 0.39	1.88 ^b^ ± 0.18	2.23 ^b^ ± 0.24	2.30 ^b^ ± 0.33
	2	0.98 ^a^ ± 0.15	1.03 ^a^ ± 0.35	1.58 ^a^ ± 0.30	2.18 ^b^ ± 0.18	2.15 ^b^ ± 0.27
	3	0.99 ^a^ ± 0.11	1.29 ^a^ ± 0.39	2.00 ^b^ ± 0.12	2.32 ^b^ ± 0.14	2.33 ^b^ ± 0.16
	4	0.95 ^a^ ± 0.10	1.28 ^a^ ± 0.24	1.39 ^a^ ± 0.23	1.68 ^a^ ± 0.17	1.87 ^a^ ± 0.13

^a, b^ means in the same column at species level, values with different superscript letters mean significant differences between groups (*p* < 0.05). G1 = entire floor pen covered with litter; G2 = floor pen covered with litter and having floor heating; G3 = partially (50:50)-slatted flooring including an area with litter; G4 = fully-slatted flooring with a sand bath (900 cm^2^).

**Table 5:** Prevalence of foot pad dermatitis (%) in broiler chickens and turkeys kept on different types of flooring at the end of the experiment.

Scores	Type of Flooring							
	Broiler				Turkey			
	G1 (*n* = 108)	G2 (*n* = 106)	G3 (*n* = 108)	G4 (*n* = 107)	G1 (*n* = 108)	G2 (*n* = 108)	G3 (*n* = 108)	G4 (*n* = 107)
0-	62 ^a^	64 ^a^	42 ^a^	44 ^a^	0	0	0	1
1-	38 ^a^	36 ^a^	58 ^a^	56 ^a^	1 ^b^	4 ^b^	0 ^b^	15 ^a^
2-	0	0	0	0	70 ^a^	78 ^a^	68 ^a^	80 ^a^
3-	0	0	0	0	29 ^ab^	18 ^ab^	32 ^a^	4 ^b^
4-	0	0	0	0	0	0	0	0

^a, b^ frequency in the same row at species level; values with different superscript letters mean significant differences between groups (*p* < 0.05). G1 = entire floor pen covered with litter; G2 = floor pen covered with litter and having floor heating; G3 = partially (50:50)-slatted flooring including an area with litter; G4 = fully-slatted flooring with a sand bath (900 cm^2^).
